# Night Jumps with Equipment and Self-Reported Musculoskeletal Problems in Military Parachutists: A Cross-Sectional Study

**DOI:** 10.3390/jfmk11030346

**Published:** 2026-08-31

**Authors:** Daniel Sanz-Blanco, Vicente Javier Clemente-Suárez

**Affiliations:** 1 Plana Mayor, Batallón de Zapadores VI de Paracaidistas, Brigada «Almogávares» VI de Paracaidistas, Base «Príncipe», Paracuellos de Jarama, 28860 Madrid, Spain; 2 Centro de Estudios de Combate Aplicado, 45007 Toledo, Spain; 3 Grupo de Investigación en Cultura, Educación y Sociedad, Universidad de la Costa, Barranquilla 080002, Colombia; 4 Department of Sport Sciences, Faculty of Sport and Health Sciences, Fit Generation Research Institute, AD500 Andorra la Vella, Andorra

**Keywords:** military parachuting, musculoskeletal problems, night jumps, load carriage, military personnel

## Abstract

**Background and Objectives**: Military parachuting combines landing impact, carried load, and restricted visual information. This study examined the concurrent associations of two primary task exposures—recent jump volume and participation in at least one night jump with equipment—with self-reported parachuting-related musculoskeletal problems; individual characteristics were treated as adjustment variables or exploratory factors. **Methods**: A cross-sectional study was conducted in 105 parachutists from the Sapper Battalion of the Spanish Parachute Brigade. Exposure and outcome were both recalled for the same preceding 12 months, and their temporal sequence was not recorded. Prevalence was estimated with Wilson 95% confidence intervals, and the two primary exposures were entered simultaneously in Firth bias-reduced logistic regression adjusted for age and body mass index. A modified Poisson model with robust standard errors estimated adjusted prevalence ratios as a sensitivity analysis. **Results**: Forty-eight participants (45.7%) reported at least one parachuting-related musculoskeletal problem during the preceding year, most frequently at the knee, low back, and upper back or neck. Problems were reported by 79.4% of participants who had performed night jumps with equipment and by 29.6% of those who had not. Night jumps with equipment were associated with the outcome (odds ratio 7.13, 95% confidence interval 2.71–20.66), whereas recent jump volume was not. The adjusted prevalence ratio for the combined exposure was 2.44 (95% confidence interval 1.63–3.64). **Conclusions**: Night jumps with equipment showed a concurrent association with self-reported musculoskeletal problems, but the design cannot establish whether the exposure preceded the problem or separate the contributions of darkness and equipment. The findings should be treated as hypothesis-generating for prospective, jump-level surveillance.

## 1. Introduction

Military parachuting, particularly static-line operations, is a safety-critical human-system task in which musculoskeletal burden may arise from the interaction between the parachutist, the parachute system, carried equipment, environmental conditions, and the landing response. Ground impact is the predominant acute injury mechanism, and the lower extremities and spine account for a substantial proportion of recorded cases [1,2]. Injury incidence nevertheless varies across aircraft, parachute systems, terrain, and meteorological conditions, indicating that landing cannot be evaluated independently of its task context [3,4]. Military parachuting therefore combines high mechanical loading, constrained movement, limited preparation time, and variable sensory information.

Night jumping while carrying equipment is especially relevant because it combines two recognized sources of task difficulty. Experimental and observational evidence suggests that restricted visual information can alter preparation for ground contact, while carried equipment increases total system mass and may constrain landing movement. Large military datasets have associated darkness and combat loads with greater injury occurrence [2,5], and a systematic review identified both factors among the most consistent correlates of injury during static-line airborne activities [3]. Experimental work further shows that combat equipment alters knee kinematics and increases vertical ground-reaction forces during landing tasks [6], while load carriage produces systematic changes in trunk and lower-limb mechanics [7,8]. Accordingly, the study considered two complementary primary task-exposure variables: recent jump volume and participation in at least one night jump with equipment. The latter represents a discrete operational condition in which restricted visual information and carried load coexist, whereas recent jump volume captures cumulative exposure. The participant-level loaded-night indicator does not quantify condition-specific exposure frequency and cannot identify the independent contribution of darkness or equipment.

Parachuting demands also vary across personnel. Experience modifies acute psychophysiological responses to military jumps [9,10,11,12], while previous injury, anthropometric characteristics, and physical capacity have shown heterogeneous associations with musculoskeletal injury in military cohorts [13,14]. Sleep may affect recovery [15,16]. These individual, training, sleep, and psychological characteristics were not prespecified confounders of the primary exposure–outcome model in the present study; they were examined separately as exploratory characteristics.

Most military parachuting research has focused on acute injuries recorded at the drop zone or through medical surveillance during specific training periods. Such approaches may not capture pain, functional limitation, or other musculoskeletal problems that do not result in immediate medical attendance or time loss. The specific gap addressed here was the participant-level association between a combined loaded-night exposure marker and 12-month self-reported regional musculoskeletal problems in a specialized Spanish military parachuting cohort while accounting for recent jump volume. The two primary exposures were the number of jumps completed during the preceding 12 months, analyzed continuously, and participation in at least one night jump with equipment. The primary aim was to estimate their concurrent associations with self-reported parachuting-related musculoskeletal problems. Physical performance, training, sleep, and psychological variables were exploratory. We hypothesized that both greater recent jump volume and the combined loaded-night condition would be associated with a higher prevalence of musculoskeletal problems, while recognizing that the cross-sectional recall design could not establish temporal order.

## 2. Materials and Methods

### 2.1. Study Design and Setting

A cross-sectional observational study was conducted in the Sapper Battalion of the Spanish Parachute Brigade (BRIPAC), Spain. Electronic questionnaire responses were recorded between 21 January and 10 April 2026, and the field-based physical assessment was conducted in March 2026. Participants were recruited through convenience sampling because access to this specialized military unit was restricted and the accessible population was limited. Battalion personnel who were available, met the eligibility criteria, and could complete the assessment during the study period were invited. The recruitment log did not retain the total number of eligible personnel, individuals approached, or personnel declining participation; therefore, a participation rate cannot be reconstructed. No a priori recruitment target was used because the accessible population was fixed. Sample adequacy was evaluated through estimation precision and model information: with n = 105, the maximum approximate half-width of a 95% confidence interval for a prevalence near 50% is 9.4 percentage points, and the 48 primary-outcome events provided 12 events per parameter for the four-variable principal model. Firth estimation was selected to reduce small-sample and sparse-cell bias. Reporting followed the Strengthening the Reporting of Observational Studies in Epidemiology recommendations for cross-sectional studies [17].

As an association-focused sensitivity assessment, a two-group proportion approximation was calculated using the observed loaded-night exposure prevalence (32.4%; 34/105), the observed primary-outcome prevalence among unexposed participants (29.6%; 21/71), a two-sided α of 0.05, and 80% power. Under these assumptions, the available sample could detect an odds ratio of approximately 3.31 or larger for the loaded-night exposure. Detecting an odds ratio of 2.0 under the same exposure allocation and baseline outcome prevalence would require approximately 322 participants. These retrospective calculations do not account for potential loss of power from covariate adjustment or exposure/outcome misclassification and therefore indicate that the study had limited sensitivity to moderate associations; they were not used to justify recruitment prospectively.

### 2.2. Participants and Ethics

The analytical sample comprised 105 active-duty military parachutists assigned to the Sapper Battalion. Eligibility required age of at least 18 years, current service in the battalion, previous military parachuting experience, written informed consent, and a questionnaire response permitting classification of the primary musculoskeletal outcome. Participants completed a self-administered electronic questionnaire. Written consent stated that participation was voluntary and could be discontinued without adverse consequences. Questionnaire and physical-assessment records were linked through a participant-specific pseudonymized code. Records were reviewed for duplicate codes and internal inconsistencies before analysis. This study was approved by the Ethics Committee of Universidad Europea on 14 October 2024 (approval code 2024-863) and complied with the Declaration of Helsinki. Sex was recorded from the demographic questionnaire as male or female. The three women were retained because sex was not an eligibility criterion and the target cohort comprised eligible active-duty parachutists; no female-specific inference was made, and a men-only sensitivity analysis was reported. Years of parachuting experience were missing for three participants (n = 102 for that variable), but all three reported prior career jumps (6, 10, and 81, respectively), confirming that they met the previous-experience eligibility criterion. The archived study documentation did not retain the identity or rank of the person who approached each participant, so this aspect of recruitment cannot be reconstructed.

### 2.3. Parachuting Exposure

The questionnaire recorded years of parachuting experience, total career jumps, jumps performed during the preceding 12 months, automatic-opening jumps, and four jump-condition indicators: daytime without additional equipment, nighttime without additional equipment, daytime with equipment, and nighttime with equipment. The two primary task exposures were (1) the number of jumps performed during the preceding 12 months, analyzed as a continuous variable, and (2) participation in at least one night jump with equipment during the same period, coded as a binary variable. Both exposures were entered simultaneously in the principal association model. The condition variables recorded occurrence only, not the number of jumps completed under each condition. Consequently, a participant with one loaded night jump was classified identically to a participant with several such jumps, and a dose–response analysis was not possible. The four condition indicators were not mutually exclusive because a participant could report more than one type of jump. The questionnaire did not distinguish training from operational jumps. Exposure questions used the same preceding 12-month recall window as the musculoskeletal outcome questions.

### 2.4. Musculoskeletal Outcomes

Musculoskeletal problems were assessed using an original regional questionnaire informed by the Oslo Sports Trauma Research Centre (OSTRC) health-problem surveillance framework [18,19], rather than by administering the validated OSTRC-O or OSTRC-H questionnaires in their intended prospective form. The questionnaire used a 12-month retrospective period, restricted the questions to parachuting-related problems, and assessed six anatomical regions: ankle or foot, knee, hip or groin, low back or lumbar region, upper back or neck, and shoulder. For each region, it recorded the presence of a problem and its effect on participation, physical performance, and pain. The primary outcome was at least one self-reported parachuting-related musculoskeletal problem in a predefined region or a parachuting-related injury described as medically diagnosed in the open-text item. A substantial problem was defined as a problem accompanied by moderate or large reduction in participation or performance, or inability to participate. The original OSTRC questionnaires were developed for prospective repeated surveillance with a short recall period; the changes in recall period, setting and regional case definition used here were substantial, and this questionnaire was not psychometrically revalidated in this military population. The outcomes were therefore interpreted as self-reported musculoskeletal problems rather than validated OSTRC scores or clinically verified diagnoses. The questionnaire did not record the onset date of each musculoskeletal problem relative to the night jump or jumps. A participant could therefore be classified as exposed and affected even if the reported problem preceded the loaded-night exposure. Region-specific adjusted models were restricted to the knee, low back, and upper back or neck because each outcome had at least 10 events. The retained questionnaire content and classification algorithm are described in Supplementary Appendix S1.

### 2.5. Training, Sleep, and Psychological Measures

Training, sleep, and psychological measures were collected as secondary exploratory characteristics rather than as prespecified confounders of the primary model. Training variables included weekly session frequency and duration, training type, pre-jump warm-up, post-jump recovery, and self-rated fitness. Sleep was represented by items derived from the Pittsburgh Sleep Quality Index (PSQI) [20]; because the available items did not reproduce the complete validated instrument, the derived score was treated as exploratory. Perceived stress was represented by a 10-item score constructed from wording related to the Perceived Stress Scale [21] but not identical to the validated PSS-10. Anxiety symptoms were assessed with the Generalized Anxiety Disorder-7 (GAD-7) [22], depressive symptoms with the Patient Health Questionnaire-9 (PHQ-9) [23], and psychological inflexibility with the Acceptance and Action Questionnaire-II (AAQ-II) [24]. These screening measures were not interpreted as clinical diagnoses. Individual nutrition and hydration questions were excluded from the principal analyses because no validated composite index had been administered or defined before analysis.

### 2.6. Field-Based Physical Assessment

Standing horizontal-jump performance was assessed after a standardized warm-up consisting of two sets of 10 vertical jumps separated by 30 s, following the procedure previously used in Spanish military personnel [25]. The same prescribed warm-up was used for every participant who completed this assessment. Participants then performed two maximal horizontal jumps from a two-foot take-off, and the longest valid distance was retained. Maximal isometric handgrip strength was measured in the dominant hand using a TKK 5402 dynamometer (Takei Scientific Instruments, Niigata, Japan). Participants were seated with the shoulder in neutral position, the elbow flexed to 90°, and the forearm neutral, consistent with the approach used by Bustamante-Sánchez et al. [26]. Two maximal contractions were performed and the highest value was retained. Relative handgrip strength was calculated as kilograms of force divided by body mass in kilograms. Posterior-chain flexibility was assessed with two sit-and-reach attempts, retaining the highest value [27]. Peripheral oxygen saturation and pulse rate were recorded with a Beurer PO 30 finger pulse oximeter. Forced vital capacity, forced expiratory volume in one second, and peak expiratory flow were obtained with a QM-SP100 portable spirometer using two maximal maneuvers and the highest technically acceptable value. Because a full set of American Thoracic Society/European Respiratory Society repeatability procedures was not implemented, respiratory measurements were treated as field characteristics rather than diagnostic spirometry.

### 2.7. Data Completeness and Management

Source records were examined for duplicate identifiers, coding errors, implausible values and internal inconsistencies. Corrections were made only when the intended value was unambiguous from the source records and the paired anthropometric or identifier information; otherwise, the value remained missing. Time-formatted sleep durations were converted to decimal hours, entries recorded in minutes were converted to hours, and uninterpretable entries remained missing. No statistical imputation was performed. Questionnaire completeness was high for the primary exposures and outcome. Physical-test availability was lower: handgrip strength was available for 90 participants; standing horizontal-jump performance for 47; sit-and-reach performance for 42; peripheral oxygen saturation and pulse rate for 88; forced vital capacity and forced expiratory volume in one second for 93; and peak expiratory flow for 92. Standing horizontal jump (44.8% available) and sit-and-reach (40.0% available) were excluded from inferential exploratory analyses because fewer than half of participants had data and the reason for test non-availability was not recorded, creating a plausible opportunity-related or missing-not-at-random selection process. Other descriptive and univariable analyses used available observations, whereas adjusted models used complete observations for the variables included in each model. Detailed completeness and outcome-specific test availability are provided in Supplementary Tables S1 and S5.

### 2.8. Statistical Analysis

Continuous variables were summarized as mean and standard deviation, and median and interquartile range; categorical variables were summarized as frequency and percentage. Outcome prevalence was accompanied by Wilson 95% confidence intervals. Characteristics of participants reporting versus not reporting a night jump with equipment were compared descriptively using Welch *t*-tests for age and body mass index, Mann–Whitney U tests for service duration and parachuting-exposure variables, and Fisher exact tests for sex. The principal association model used Firth bias-reduced logistic regression to reduce small-sample and sparse-cell bias [28,29]. The presence of any musculoskeletal problem was the dependent variable. Night jumps with equipment and jumps during the preceding 12 months were entered simultaneously and adjusted for age and body mass index. Continuous predictors were standardized; their odds ratios therefore represent a one-standard-deviation increase. Odds ratios were reported with profile penalized-likelihood 95% confidence intervals and likelihood-ratio *p*-values. Sensitivity analyses additionally adjusted for years of parachuting experience and, separately, career jump count; used log-transformed recent jump volume; restricted the analysis to men; and used substantial musculoskeletal problems as the outcome. A further Firth model entered all four non-mutually exclusive jump-condition indicators simultaneously with recent jump volume, age, and body mass index to examine whether the combined loaded-night indicator remained associated after accounting for the other recorded conditions; this model was not interpreted as a causal decomposition of darkness and equipment. Because the primary outcome was common, a modified Poisson model with robust standard errors was also fitted to estimate adjusted prevalence ratios. Separate adjusted models examined knee, low-back, and upper-back or neck problems. Potential collinearity was examined using pairwise correlations and variance inflation factors; the correlation matrix and VIF values are reported in Supplementary Table S6. Physical, training, sleep and psychological variables were analyzed only as secondary exploratory factors. Standing horizontal jump and sit-and-reach were excluded from inferential testing because of low and potentially outcome-related availability. Remaining exploratory *p*-values were adjusted using the Benjamini–Hochberg false-discovery-rate procedure within defined analytical families [30], and these analyses were not used to determine the principal conclusions. Statistical tests were two-sided, with *p* < 0.05 considered statistically significant. Analyses were conducted in Python 3.11 using NumPy, SciPy, and statsmodels; Firth models used an adjusted-score algorithm with profile penalized-likelihood inference.

### 2.9. Use of Generative Artificial Intelligence

During the preparation of this work, the authors used OpenAI ChatGPT (GPT-5.6 Sol) to assist with language editing, document organization, and figure preparation. The authors reviewed and edited the content and take full responsibility for the content of the manuscript.

## 3. Results

The analytical sample included 105 participants, of whom 102 were men and three were women. Mean age was 27.80 years (SD 8.06), mean military-service duration was 6.98 years (SD 7.92), and mean body mass index was 25.45 kg/m^2^ (SD 2.70). The median number of career jumps was 12 (interquartile range 8–21), and the median number completed during the preceding 12 months was 3 (interquartile range 1–6). Thirty-four participants (32.4%) reported at least one night jump with equipment during the same period. Compared with the 71 participants without this exposure, the 34 exposed participants were younger (25.79 ± 5.55 vs. 28.76 ± 8.90 years; *p* = 0.040), had a higher median career jump count (15 [11–25.75] vs. 11 [6–20]; *p* = 0.034), and had completed more jumps during the preceding 12 months (5 [4–6] vs. 1 [0–6]; *p* < 0.001). No statistically detectable group differences were observed for sex, service duration, body mass index, or years of parachuting experience. These comparisons are descriptive and unadjusted. Core participant and exposure characteristics by loaded-night exposure are presented in Table 1.

Forty-eight participants reported at least one parachuting-related musculoskeletal problem during the preceding 12 months, corresponding to 45.7% (95% confidence interval 36.5–55.2%). Twenty-seven reported a substantial problem (25.7%, 95% confidence interval 18.3–34.8%). The most frequently affected regions were the knee (21.0%), low back or lumbar region (20.0%) and upper back or neck (18.1%); prevalence for the remaining regions was below 7% (Table 2).

The archived analytical dataset retained the composite primary-outcome classification but did not retain a separate indicator identifying whether a participant was classified as positive exclusively because of a medically diagnosed parachuting-related injury described in the open-text field. Consequently, the number of the 48 positive cases attributable solely to that criterion cannot be reconstructed from the archived analytical dataset.

**Figure 1:**
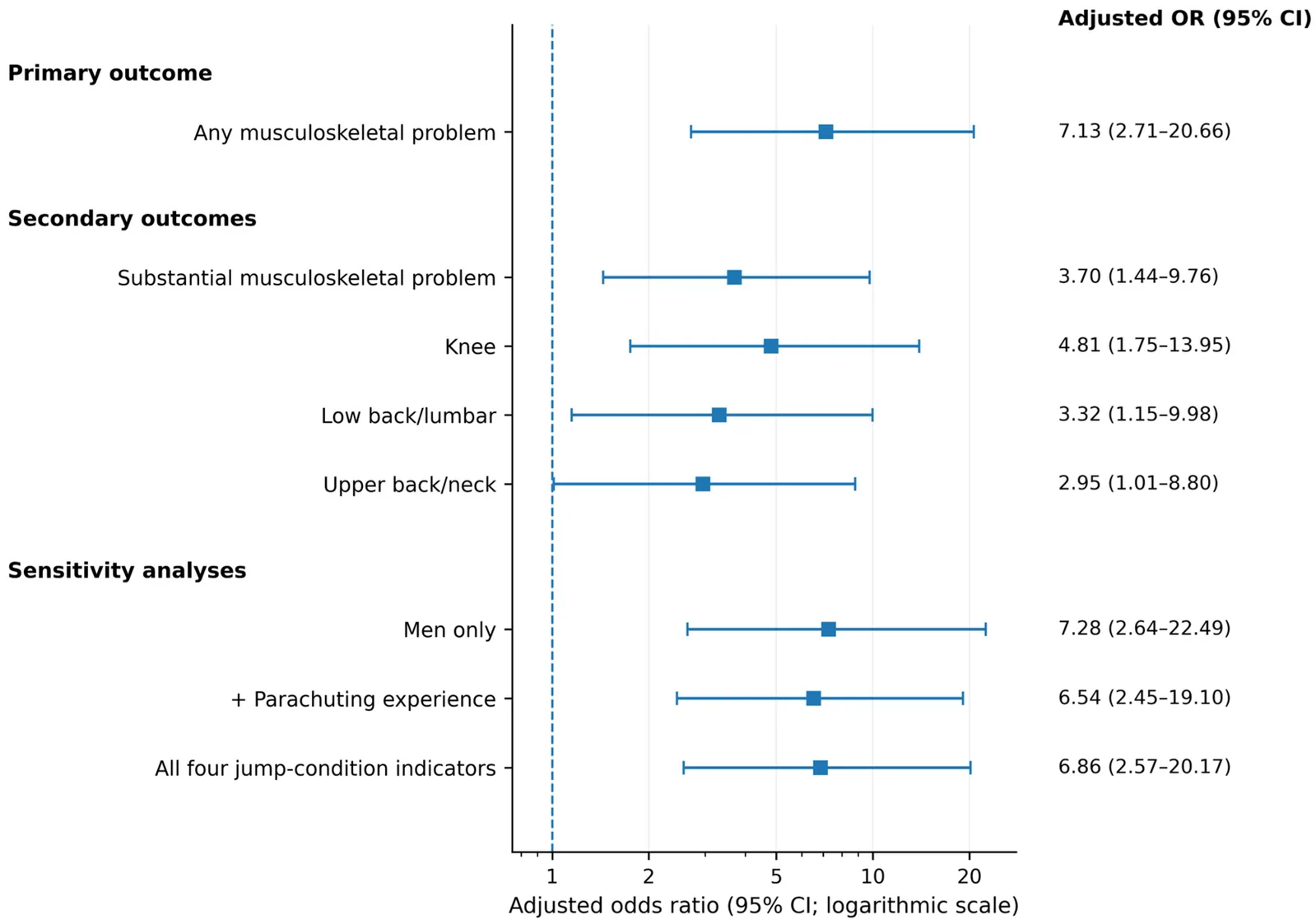
Adjusted associations between night jumps with equipment and self-reported musculoskeletal outcomes and sensitivity analyses. Squares indicate adjusted odds ratios, and horizontal lines indicate profile penalized-likelihood 95% confidence intervals. The primary, substantial-problem, and region-specific models included night jumps with equipment, recent jump volume, age, and body mass index. Sensitivity estimates displayed in the revised figure include the men-only model, additional adjustment for years of parachuting experience, and the model containing all four recorded jump-condition indicators simultaneously. The dashed line indicates an odds ratio of 1. The modified Poisson sensitivity estimate is reported separately in the Results and Supplementary Table S3 because it is expressed as an adjusted prevalence ratio rather than an odds ratio.

In the principal adjusted model, night jumps with equipment were associated with higher odds of reporting any musculoskeletal problem (odds ratio 7.13, 95% confidence interval 2.71–20.66; *p* < 0.001), whereas the number of jumps performed during the preceding 12 months was not independently associated (odds ratio per standard deviation 1.03, 95% confidence interval 0.70–2.20; *p* = 0.899). Age showed an inverse coefficient in the principal model, while body mass index was not associated (Table 3). Because the outcome prevalence was 45.7%, the modified Poisson sensitivity model was used to express the association on the prevalence-ratio scale; the adjusted prevalence ratio for night jumps with equipment was 2.44 (95% confidence interval 1.63–3.64; *p* < 0.001). The estimate for night jumps with equipment remained similar after additional adjustment for parachuting experience and career jumps, in the men-only analysis, and when a substantial problem was used as the outcome. In the sensitivity model containing all four recorded jump-condition indicators simultaneously, the night-with-equipment indicator remained associated with the outcome (odds ratio 6.86, 95% confidence interval 2.57–20.17; *p* < 0.001), whereas daytime without equipment, nighttime without equipment, and daytime with equipment were not individually associated. Because these indicators were non-mutually exclusive and condition-specific jump counts were unavailable, this model does not separate the independent effects of darkness and equipment. Variance inflation factors in the principal model ranged from 1.07 to 1.17, with no concerning collinearity. Region-specific estimates were positive for the knee, low back, and upper back or neck but were based on only 19–22 events per region and had wide confidence intervals; they are therefore secondary and imprecise. The adjusted estimates for the principal, regional, and sensitivity analyses are summarized in Figure 1, with exact model results and additional sensitivity analyses in the Supplementary Materials.

**Figure 2:**
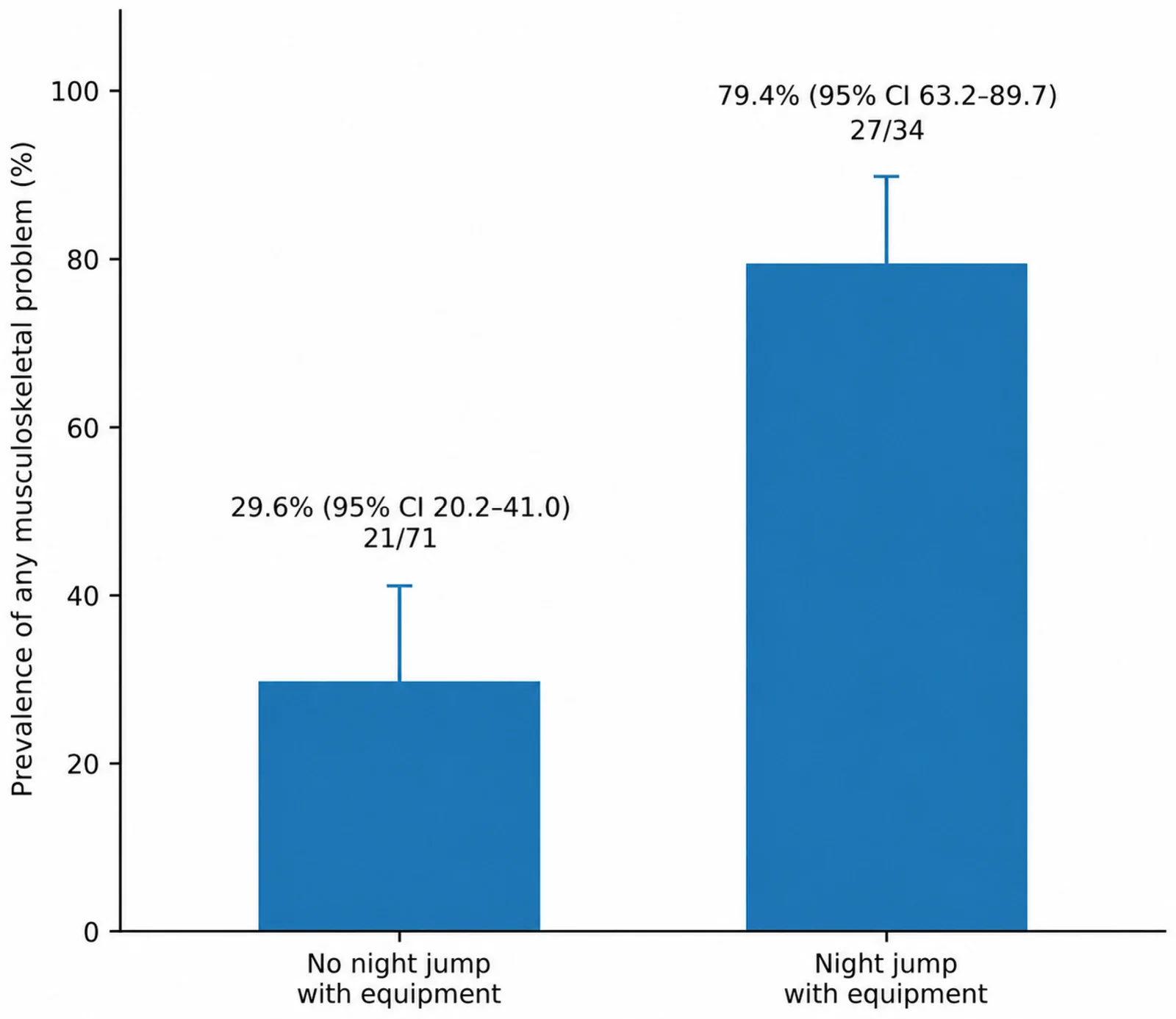
Prevalence of any self-reported parachuting-related musculoskeletal problem according to exposure to night jumps with equipment. Bars show percentages, error bars show Wilson 95% confidence intervals, and fractions indicate participants with the outcome divided by the number in each exposure group.

Thirty-four participants had performed at least one night jump with equipment. Musculoskeletal problems were reported by 27 of these participants (79.4%, 95% confidence interval 63.2–89.7%) and by 21 of the 71 participants without this exposure (29.6%, 95% confidence interval 20.2–41.0%). This unadjusted prevalence contrast is shown in Figure 2.

Secondary exploratory analyses were deliberately separated from the primary task-exposure models. Higher relative handgrip strength showed a positive univariable association with the overall outcome after false-discovery-rate correction, but this finding was based on 90 available measurements and was not interpreted causally. No sleep or psychological variable remained associated after correction for multiple comparisons. Standing horizontal jump and sit-and-reach were removed from inferential exploratory analyses because of their low completeness. Horizontal-jump availability differed between participants without and with a musculoskeletal problem (19/57, 33.3%, versus 28/48, 58.3%; Fisher *p* = 0.012), whereas sit-and-reach availability showed a smaller, non-significant difference (18/57, 31.6%, versus 24/48, 50.0%; *p* = 0.072). Availability of handgrip, pulse oximetry, and spirometric measurements did not differ detectably by outcome status. Because reasons for non-testing were not recorded, the horizontal-jump pattern is compatible with opportunity-related or outcome-related missingness and precludes reliable inference from that test. The complete retained exploratory results and availability analyses are reported in Supplementary Tables S4 and S5 and did not alter the principal interpretation.

## 4. Discussion

The principal finding was a concurrent association between reporting at least one night jump with equipment and reporting a parachuting-related musculoskeletal problem within the same 12-month retrospective window. The association remained after adjustment for age, body mass index, and recent jump volume, after additional control for parachuting experience and career jumps, in the men-only analysis and when substantial problems were considered. On the prevalence-ratio scale, exposed participants had an adjusted prevalence 2.44 times that of unexposed participants. This estimate must not be read temporally: the questionnaire did not establish whether the loaded night jump preceded the onset of the musculoskeletal problem, so reverse temporal ordering is possible. Recent jump volume did not remain independently associated. Thus, the data supported the loaded-night component of the hypothesis but not the proposed association with greater recent jump volume. The study cannot determine whether darkness, carried equipment, their combination, or characteristics of personnel assigned to such jumps account for the observed association.

The 12-month prevalence of any musculoskeletal problem was 45.7%, and approximately one quarter of participants reported a moderate or greater effect on participation or performance. These values cannot be compared directly with per-jump injury rates because the denominators, time frames, and case definitions differ. Prospective military surveillance has reported approximately 2.5–35.5 injuries per 1000 jumps [2,4,31], while an elite Ranger battalion reported injuries in 2.2% of static-line jumps [32]. The present study instead captured pain, functional limitation, or a reported diagnosis at any time during the preceding year, including problems not resulting in immediate medical attendance. This interpretation is consistent with evidence that paratroopers have greater odds of both acute and cumulative musculoskeletal injury than non-paratrooper soldiers [33]. The prevalence therefore represents accumulated musculoskeletal burden rather than injury probability per descent.

The concurrent association with loaded night jumps is compatible with prior military parachuting evidence, but the present data do not test darkness and equipment as independent causal components. Night conditions and combat loads were independent injury-related factors in more than 23,000 jumps [2] and remained associated in a later analysis of more than 131,000 T-10 and T-11 descents [4]. Equipment carriage and night descent were also identified across 34,236 descents [5], and an elite Ranger battalion experienced a higher injury rate and approximately 2.5 times as many severe injuries at night [32]. A systematic review confirmed night jumping and additional equipment among the most reproducible correlates of static-line parachuting injury [3], while Thai military data showed estimates in the same direction, although imprecisely [31]. The larger odds ratio observed here may reflect the combined binary exposure definition, differences in assignment or experience, and the cumulative self-reported outcome rather than a stronger per-jump effect; the wide confidence interval indicates substantial uncertainty in the exact magnitude.

Darkness and equipment provide a plausible biomechanical and perceptual rationale for studying the combined condition, but the mechanisms were not measured here. Visual information supports estimation of time to contact and preparatory lower-limb activation. Removing vision during experimental drop landings increased ground-reaction forces, reduced knee rotation, and increased response variability [34]. Equipment simultaneously increases mechanical demand: combat loads raised maximal vertical ground-reaction force and altered knee-flexion timing in air-assault soldiers [6]. Military load carriage also modifies trunk and lower-limb kinematics, joint moments, and muscle activation [8], while heavier loads increase forces transmitted through the lower extremities [7]. These findings make the combined exposure mechanistically plausible, but the present cross-sectional analysis cannot identify which component, if either, accounts for the observed concurrent association.

Psychophysiological evidence provides further context, although these mechanisms were not assessed as mediators. Automatic and tactical jumps produce marked cardiovascular, sympathetic, and metabolic activation, with greater heart rate, lactate, and anxiety responses in novice than experienced parachutists [9,10,11]. High-altitude jump modalities also elicit distinct autonomic and physiological responses [12]. These studies do not establish that acute stress causes musculoskeletal problems, but they demonstrate that parachuting requires simultaneous perceptual, physiological, and motor regulation. Darkness and equipment may intensify these combined demands when postural preparation and landing execution are most critical.

The anatomical distribution must be interpreted according to the broader outcome definition. Acute surveillance identifies landing as the predominant mechanism, and the lower extremity, particularly the ankle, as the most frequently injured region [1,2,35]. Here, the knee, low back, and upper back or neck predominated because the questionnaire also captured persistent or recurrent pain and functional impairment. A 12-month survey of sport skydivers similarly reported neck pain in 25%, low-back pain in 18% and thoracic pain in 10% [36]. Together with the greater overall injury odds observed in paratroopers [33], these findings support concern about cumulative, as well as acute, consequences. The regional associations are biomechanically coherent with force transmission through the lower limbs and trunk, although their estimates remain imprecise and require prospective confirmation.

Recent jump volume was associated with the outcome before adjustment but not after jump conditions and participant characteristics were considered. This differs from sport skydivers, among whom 30–90 and more than 90 annual jumps were associated with pain [36]. The current participants completed a median of only three jumps during the preceding year, limiting discrimination of cumulative loading. Moreover, a count assigns equal weight to unloaded daytime and loaded night descents despite risk variation by equipment, wind, aircraft, terrain, parachute system, and time of day [2,3,4,31]. The result does not indicate that repeated exposure is harmless, but that contextual characteristics provided more information in this cohort.

Age was inversely associated in the principal model but weakened after inclusion of parachuting experience. This should not be interpreted as a protective biological effect, because military studies have reported increasing injury risk with age [35,37], whereas limited experience with a parachute system is also relevant [3]. The positive exploratory association with relative handgrip strength is similarly unlikely to indicate harm: handgrip does not directly measure impact attenuation, stronger personnel may receive more equipment-intensive assignments, and measurements were incomplete. Reviews show heterogeneous associations for isolated fitness tests, with previous injury and task exposure generally providing more consistent information [13,14]. Sleep and psychological variables did not survive multiple-comparison correction, although effects on recovery or motor control cannot be excluded.

Annual jump totals alone may be an incomplete description of parachuting exposure. A prospective surveillance design could record each descent by equipment mass and distribution, visibility, wind, aircraft and exit type, parachute system, drop altitude, terrain or landing surface, landing quality, and immediate symptoms. The present findings do not show that altering equipment, training progression, or landing preparation would reduce musculoskeletal problems. They identify the combined loaded-night condition as a candidate exposure to test prospectively with jump-level temporal data.

This study benefits from access to a specialized airborne engineering battalion and from consistency across several sensitivity analyses. Its limitations, however, materially restrict interpretation. First, exposure and outcome were assessed retrospectively over the same 12-month period, and the questionnaire did not record whether a musculoskeletal problem began before or after the relevant night jump. The association is therefore concurrent and cannot establish temporality or causality. Second, the loaded-night primary exposure was binary: condition-specific jump counts were unavailable, so exposure frequency and dose–response could not be examined. The four jump-condition indicators were also non-mutually exclusive and did not distinguish training from operational jumps. Third, personnel assigned to loaded night jumps may differ systematically in role, tasking, experience, or operational demands. Although age, recent jump volume, parachuting experience, and career jumps were examined in adjusted or sensitivity models, residual confounding by assignment and unmeasured task characteristics remains plausible. Fourth, previous injury, individual equipment mass and load distribution, parachute type, drop altitude, wind, landing surface or terrain, aircraft, and landing technique were not available at participant or jump level. Verified study-period unit ranges for these characteristics were not retained in the research archive. Fifth, the regional questionnaire was an original instrument informed by the OSTRC framework but substantially modified to a 12-month parachuting-specific retrospective format and was not psychometrically revalidated; recall error and case-classification error are possible. Self-report in a military environment may additionally be affected by underreporting or social-desirability concerns, despite voluntary participation and pseudonymized analysis. Sixth, convenience recruitment creates opportunity bias, and the numbers eligible, approached, and declining were not retained, so selection into the study cannot be quantified. Physical-test availability was incomplete; horizontal-jump availability was associated with outcome status, and missingness could be missing-not-at-random. For this reason, horizontal jump and sit-and-reach were excluded from inferential exploratory analyses. Finally, the cohort came from one battalion and included only three women. The women were retained to preserve the eligible cohort, but the sample cannot support sex-specific inference and is primarily informative for male military parachutists. These constraints mean that the observed association should be used to design prospective surveillance, not as an individual prediction rule or evidence that loaded night jumps cause musculoskeletal problems.

The sensitivity assessment indicated limited power for moderate associations: under the observed exposure allocation and unexposed outcome prevalence, the approximate minimum detectable odds ratio was 3.31. Questionnaire completion occurred between 21 January and 10 April 2026; therefore, each participant’s 12-month retrospective window was anchored to the individual completion date, and the windows were shifted by up to 79 days across respondents rather than referring to one identical calendar period. In addition, the archived analytical dataset did not retain a separate indicator identifying cases classified as positive exclusively from the open-text medically diagnosed injury criterion. The literal wording of the questionnaire items was not retained, and therefore the instrument is not reproducible in future studies.

## 5. Conclusions

In this cohort, reporting at least one night jump with equipment and reporting a parachuting-related musculoskeletal problem were concurrently associated within the same preceding 12-month recall period. The association was also observed for substantial problems and in secondary knee and spinal analyses, whereas recent jump volume did not retain an independent association after adjustment. Because temporal order was not recorded, condition-specific jump frequency was unavailable, and the design could not separate darkness from equipment carriage, the findings do not support causal inference. Prospective jump-level studies that record exposure dose, timing, equipment, environment, and incident symptoms are required to determine whether the combined condition predicts subsequent musculoskeletal problems.

## Figures and Tables

**Table 1:** Participant characteristics according to reported night-jump-with-equipment exposure.

Variable	Overall	No Night Jump with Equipment (n = 71)	Night Jump with Equipment (n = 34)	*p*-Value
Age, years	27.80 ± 8.06	28.76 ± 8.90	25.79 ± 5.55	0.040
Men	102 (97.1%)	69 (97.2%)	33 (97.1%)	1.000
Women	3 (2.9%)	2 (2.8%)	1 (2.9%)	1.000
Years of military service	4.00 [2.00–8.00]	5.00 [2.00–9.50]	3.00 [2.00–5.88]	0.390
Body mass index, kg/m^2^	25.45 ± 2.70	25.67 ± 2.89	24.99 ± 2.24	0.185
Parachuting experience, years	2.75 [1.00–5.75] (n = 102)	3.00 [1.00–6.00] (n = 69)	2.00 [1.00–3.00] (n = 33)	0.256
Career jumps	12.00 [8.00–21.00]	11.00 [6.00–20.00]	15.00 [11.00–25.75]	0.034
Jumps in previous 12 months	3.00 [1.00–6.00]	1.00 [0.00–6.00]	5.00 [4.00–6.00]	<0.001

Values are mean ± standard deviation, median [interquartile range], or number (percentage), as appropriate. Group comparisons used Welch *t* tests for age and body mass index, Mann–Whitney U tests for service duration and parachuting-exposure variables, and Fisher exact tests for sex. The *p*-values are descriptive and unadjusted. Years of parachuting experience were missing for three participants. SD, standard deviation; IQR, interquartile range.

**Table 2:** Prevalence of self-reported parachuting-related musculoskeletal problems during the preceding 12 months.

Outcome	Events	Total	Prevalence, % (95% CI)
Any musculoskeletal problem	48	105	45.7 (36.5–55.2)
Substantial musculoskeletal problem	27	105	25.7 (18.3–34.8)
Ankle/foot	4	105	3.8 (1.5–9.4)
Knee	22	105	21.0 (14.3–29.7)
Hip/groin	6	105	5.7 (2.6–11.9)
Low back/lumbar	21	105	20.0 (13.5–28.6)
Upper back/neck	19	105	18.1 (11.9–26.5)
Shoulder	7	105	6.7 (3.3–13.1)

Confidence intervals were calculated using the Wilson method. CI, confidence interval.

**Table 3:** Firth bias-reduced logistic regression for any self-reported musculoskeletal problem.

Predictor	Adjusted OR (95% CI)	*p*-Value
Jumps in previous 12 months, per SD	1.03 (0.70–2.20)	0.899
Night jumps with equipment	7.13 (2.71–20.66)	<0.001
Age, per SD	0.63 (0.37–0.99)	0.047
Body mass index, per SD	0.99 (0.63–1.53)	0.963

Continuous predictors were standardized; their odds ratios represent a one-standard-deviation increase. OR, odds ratio; CI, confidence interval; SD, standard deviation. Confidence intervals are profile penalized-likelihood intervals; unlike Wald intervals, they are likelihood-based and are not constrained to be symmetric around the point estimate on the log-odds scale.

## Data Availability

The data are not publicly available, because they concern active-duty military personnel and are subject to privacy and institutional restrictions. Requests for access may be directed to the corresponding author and require the relevant institutional and military authorizations.

## Supplementary Materials

The following supporting information can be downloaded at https://www.mdpi.com/article/10.3390/jfmk11030346/s1, Table S1: Detailed descriptive characteristics and data completeness; Table S2: Sensitivity and region-specific Firth logistic regression models; Table S3: Adjusted prevalence-ratio sensitivity model; Table S4: Exploratory univariable associations with any musculoskeletal problem; Table S5: Availability of field physical tests by primary-outcome status; Table S6: Principal-model predictor correlations and variance inflation factors; Table S7: STROBE cross-sectional reporting checklist; Appendix S1: Retained questionnaire content and outcome-classification algorithm; Figure S1: Available analytical participant flow.
