# Supplementary material for: Night Jumps with Equipment and Self-Reported Musculoskeletal Problems in Military Parachutists: A Cross-Sectional Study
Source: J Funct Morphol Kinesiol. 2026 Aug 31;11(3):346. doi: 10.3390/jfmk11030346 (PMC13608204; doi:10.3390/jfmk11030346)
Supplement: Supplementary file 1 [file jfmk-11-00346-s001.zip › jfmk-4514500-supplementary.pdf]

# Supplementary Material

*Night jumps with equipment and self-reported musculoskeletal problems in military parachutists: A cross-sectional study*

## Supplementary Table S1. Detailed descriptive characteristics and data completeness

| Variable | n | Mean ± SD | Median [IQR] |
|---|---|---|---|
| Age, years | 105 | 27.80 ± 8.06 | 26.00 [23.00-31.00] |
| Years of military service | 105 | 6.98 ± 7.92 | 4.00 [2.00-8.00] |
| Body mass index, kg/m² | 105 | 25.45 ± 2.70 | 25.06 [23.74-26.85] |
| Parachuting experience, years | 102 | 4.29 ± 5.04 | 2.75 [1.00-5.75] |
| Career jumps | 105 | 21.68 ± 36.32 | 12.00 [8.00-21.00] |
| Jumps in previous 12 months | 105 | 4.04 ± 5.63 | 3.00 [1.00-6.00] |
| Training sessions/week | 103 | 5.64 ± 2.30 | 5.00 [5.00-6.00] |
| Weekly training volume, min/week | 103 | 304.85 ± 240.81 | 300.00 [170.00-400.00] |
| Handgrip strength, kg | 90 | 52.89 ± 9.33 | 52.60 [47.65-58.70] |
| Relative handgrip strength, kg/kg | 90 | 0.68 ± 0.11 | 0.67 [0.62-0.74] |
| Standing horizontal jump, cm | 47 | 160.79 ± 15.16 | 160.00 [150.50-170.50] |
| Sit-and-reach, cm | 42 | 17.81 ± 9.14 | 18.00 [13.00-24.38] |
| Peripheral oxygen saturation, % | 88 | 97.17 ± 1.15 | 97.00 [97.00-98.00] |
| Pulse rate, beats/min | 88 | 72.14 ± 16.99 | 72.50 [63.75-82.00] |
| Forced vital capacity, L | 93 | 4.79 ± 0.78 | 4.79 [4.35-5.27] |
| Forced expiratory volume in 1 s, L | 93 | 3.94 ± 0.68 | 3.95 [3.58-4.31] |
| Peak expiratory flow, L/s | 92 | 9.03 ± 2.55 | 9.42 [7.81-10.86] |
| PSQI-derived score | 91 | 5.44 ± 2.62 | 5.00 [4.00-7.00] |
| PSS-related item score | 105 | 12.44 ± 6.27 | 12.00 [8.00-16.00] |
| GAD-7 total | 105 | 2.76 ± 3.23 | 2.00 [0.00-4.00] |
| PHQ-9 total | 105 | 2.50 ± 3.20 | 1.00 [0.00-4.00] |
| AAQ-II total | 105 | 10.49 ± 4.70 | 8.00 [7.00-12.00] |

PSQI, Pittsburgh Sleep Quality Index; PSS, Perceived Stress Scale; GAD-7, Generalized Anxiety Disorder-7; PHQ-9, Patient Health Questionnaire-9; AAQ-II, Acceptance and Action Questionnaire-II. Horizontal jump and sit-and-reach are retained descriptively only because of low availability.

## Supplementary Table S2. Sensitivity and region-specific Firth logistic regression models

| Analysis/outcome | Exposure | Adjusted OR (95% CI) | p value |
|---|---|---|---|
| Additional adjustment for experience | Recent jumps, per SD | 1.08 (0.71-3.12) | 0.775 |
| Additional adjustment for experience | Night jumps with equipment | 6.54 (2.45-19.10) | <0.001 |
| Log-transformed recent jumps | Recent jumps, log scale | 1.40 (0.78-2.58) | 0.264 |
| Log-transformed recent jumps | Night jumps with equipment | 5.63 (2.08-16.72) | <0.001 |
| Substantial problem | Recent jumps, per SD | 0.92 (0.50-1.33) | 0.641 |
| Substantial problem | Night jumps with equipment | 3.70 (1.44-9.76) | 0.006 |
| Men only | Recent jumps, per SD | 1.05 (0.69-3.13) | 0.863 |
| Men only | Night jumps with equipment | 7.28 (2.64-22.49) | <0.001 |
| Knee | Recent jumps, per SD | 0.98 (0.56-1.43) | 0.908 |
| Knee | Night jumps with equipment | 4.81 (1.75-13.95) | 0.002 |
| Low back/lumbar | Recent jumps, per SD | 1.33 (0.90-4.04) | 0.165 |
| Low back/lumbar | Night jumps with equipment | 3.32 (1.15-9.98) | 0.026 |
| Upper back/neck | Recent jumps, per SD | 1.01 (0.58-1.48) | 0.948 |
| Upper back/neck | Night jumps with equipment | 2.95 (1.01-8.80) | 0.047 |
| Additional adjustment for career jumps | Recent jumps, per SD | 0.99 (0.64-2.27) | 0.955 |
| Additional adjustment for career jumps | Night jumps with equipment | 7.12 (2.71-20.69) | <0.001 |
| Additional adjustment for career jumps | Career jumps, per SD | 1.10 (0.65-1.66) | 0.626 |
| All four condition indicators | Recent jumps, per SD | 1.02 (0.70-2.39) | 0.934 |
| All four condition indicators | Day without equipment | 0.53 (0.14-1.93) | 0.341 |
| All four condition indicators | Night without equipment | 1.60 (0.45-5.93) | 0.470 |
| All four condition indicators | Day with equipment | 1.82 (0.67-5.10) | 0.237 |
| All four condition indicators | Night with equipment | 6.86 (2.57-20.17) | <0.001 |

All models used Firth bias-reduced logistic regression. The primary specification adjusted for age and body mass index and included recent jump volume and night jumps with equipment. The four-condition model entered the four non-mutually-exclusive condition indicators simultaneously with recent jump volume, age and body mass index. Because the indicators overlap and do not encode condition-specific frequency, this model does not decompose the causal contributions of darkness and equipment. Confidence intervals are profile penalised-likelihood intervals and need not be symmetric around the point estimate on the log-odds scale.

## Supplementary Table S3. Adjusted prevalence-ratio sensitivity model

| Predictor | Adjusted PR (95% CI) | p value |
|---|---|---|
| Night jumps with equipment | 2.44 (1.63-3.64) | <0.001 |
| Jumps in previous 12 months, per SD | 1.03 (0.96-1.10) | 0.392 |
| Age, per SD | 0.76 (0.62-0.93) | 0.009 |
| Body mass index, per SD | 0.98 (0.81-1.19) | 0.858 |

Adjusted prevalence ratios were estimated with a modified Poisson model using robust HC0 standard errors. Continuous predictors were standardised. PR, prevalence ratio; CI, confidence interval; SD, standard deviation.

## Supplementary Table S4. Exploratory univariable associations with any musculoskeletal problem

| Family | Variable | n | OR (95% CI) | p value | FDR q |
|---|---|---|---|---|---|
| Parachuting exposure | Night jumps with equipment | 105 | 8.61 (3.28-22.58) | <0.001 | <0.001 |
| Parachuting exposure | Jumps in previous 12 months, per SD | 105 | 2.09 (1.04-4.21) | 0.020 | 0.099 |
| Parachuting exposure | Day jumps with equipment | 105 | 2.38 (1.02-5.59) | 0.039 | 0.129 |
| Parachuting exposure | Parachuting experience, per SD | 102 | 0.75 (0.48-1.16) | 0.155 | 0.388 |
| Parachuting exposure | Water jump | 105 | 1.75 (0.67-4.59) | 0.245 | 0.490 |
| Parachuting exposure | Automatic jumps, per SD | 92 | 0.92 (0.60-1.40) | 0.673 | 0.971 |
| Parachuting exposure | More than one parachute system | 105 | 1.17 (0.54-2.54) | 0.680 | 0.971 |
| Parachuting exposure | Night jumps without equipment | 105 | 1.07 (0.47-2.46) | 0.871 | 0.995 |
| Parachuting exposure | Career jumps, per SD | 105 | 1.02 (0.69-1.50) | 0.904 | 0.995 |
| Parachuting exposure | Day jumps without equipment | 105 | 1.00 (0.44-2.26) | 0.996 | 0.995 |
| Physical/training | Relative handgrip strength, per SD | 90 | 2.03 (1.22-3.39) | 0.002 | 0.027 |
| Physical/training | Age, per SD | 105 | 0.56 (0.35-0.90) | 0.007 | 0.039 |
| Physical/training | Self-rated fitness, per SD | 105 | 1.28 (0.86-1.91) | 0.206 | 0.481 |
| Physical/training | Strength training | 105 | 0.50 (0.15-1.64) | 0.230 | 0.481 |
| Physical/training | Body mass index, per SD | 105 | 0.83 (0.56-1.24) | 0.345 | 0.481 |
| Physical/training | Weekly training volume, per SD | 103 | 1.18 (0.80-1.75) | 0.387 | 0.481 |
| Physical/training | Pre-jump warm-up frequency, per SD | 105 | 1.18 (0.80-1.74) | 0.393 | 0.481 |
| Physical/training | Plyometric training | 105 | 1.21 (0.36-4.04) | 0.746 | 0.747 |
| Physical/training | Post-jump recovery frequency, per SD | 105 | 0.94 (0.64-1.38) | 0.747 | 0.747 |
| Sleep/psychological | Sleep efficiency, per SD | 95 | 0.82 (0.54-1.24) | 0.310 | 0.977 |
| Sleep/psychological | Sleep duration, per SD | 97 | 0.89 (0.59-1.33) | 0.545 | 0.977 |
| Sleep/psychological | AAQ-II, per SD | 105 | 1.09 (0.74-1.60) | 0.655 | 0.977 |
| Sleep/psychological | PSQI-derived score, per SD | 91 | 1.04 (0.68-1.57) | 0.866 | 0.977 |
| Sleep/psychological | GAD-7, per SD | 105 | 0.97 (0.66-1.43) | 0.889 | 0.977 |
| Sleep/psychological | PHQ-9, per SD | 105 | 0.98 (0.67-1.45) | 0.931 | 0.977 |
| Sleep/psychological | PSS-related item score, per SD | 105 | 0.99 (0.68-1.46) | 0.977 | 0.977 |

Continuous predictors were standardised. Binary predictors are presented relative to absence of the exposure. Analyses were exploratory and unadjusted. Standing horizontal jump and sit-and-reach were excluded from inferential analysis because fewer than half of participants had available measurements and availability could be outcome-related. FDR, false-discovery rate; OR, odds ratio; CI, confidence interval.

## Supplementary Table S5. Availability of field physical tests by primary-outcome status

| Assessment | No problem (n = 57), available n (%) | Problem (n = 48), available n (%) | Fisher p |
|---|---|---|---|
| Standing horizontal jump | 19 (33.3%) | 28 (58.3%) | 0.012 |
| Sit-and-reach | 18 (31.6%) | 24 (50.0%) | 0.072 |
| Handgrip strength | 49 (86.0%) | 41 (85.4%) | 1.000 |
| Peripheral oxygen saturation / pulse rate | 47 (82.5%) | 41 (85.4%) | 0.793 |
| Forced vital capacity / FEV1 | 50 (87.7%) | 43 (89.6%) | 1.000 |
| Peak expiratory flow | 50 (87.7%) | 42 (87.5%) | 1.000 |

Availability refers to the presence of a usable measurement. Reasons for non-testing were not retained. The association between horizontal-jump availability and outcome status makes a complete-case association difficult to interpret; horizontal jump and sit-and-reach were therefore excluded from inferential exploratory analyses. FEV1, forced expiratory volume in one second.

## Supplementary Table S6. Principal-model predictor correlations and variance inflation factors

| Predictor | Recent jumps | Night + equipment | Age | BMI | VIF |
|---|---|---|---|---|---|
| Recent jumps | 1.000 | 0.340 | -0.171 | -0.177 | 1.17 |
| Night + equipment | 0.340 | 1.000 | -0.173 | -0.120 | 1.15 |
| Age | -0.171 | -0.173 | 1.000 | 0.199 | 1.08 |
| BMI | -0.177 | -0.120 | 0.199 | 1.000 | 1.07 |

Entries are pairwise correlations among the four variables used in the principal model. All variance inflation factors were below 1.2. BMI, body mass index; VIF, variance inflation factor.

## Appendix S1. Retained questionnaire content and outcome-classification algorithm

**Instrument status.** The study used an original regional questionnaire informed by the OSTRC health-problem surveillance framework. It was not the validated OSTRC-O or OSTRC-H questionnaire and was not psychometrically revalidated after adaptation. The respondent-facing wording of every item was not retained in the final analytical archive; the content below therefore reports the operational content and coding rules that can be verified from the archived study records rather than claiming a verbatim reproduction.

- **Recall period:** The preceding 12 months was used for both parachuting exposure and musculoskeletal-problem reporting.
- **Parachuting exposure:** Total career jumps, jumps in the preceding 12 months, automatic-opening jumps, and occurrence of daytime without equipment, night-time without equipment, daytime with equipment, night-time with equipment, and water-jump conditions were recorded. The four day/night × equipment variables were occurrence indicators. The number of jumps within each condition was not recorded, and training versus operational jumps were not distinguished.
- **Primary exposure:** Night jump with equipment = 1 when the participant reported at least one such jump in the preceding 12 months; otherwise = 0.
- **Anatomical regions:** Ankle/foot; knee; hip/groin; low back/lumbar region; upper back/neck; shoulder.
- **Regional problem content:** For each region, archived variables recorded whether a parachuting-related musculoskeletal problem was present and its effect on participation, physical performance and pain.
- **Open-text injury item:** The questionnaire allowed a parachuting-related injury described by the participant as medically diagnosed to be entered in an open-text field.
- **Primary outcome:** Any musculoskeletal problem = 1 when at least one predefined region was positive or a parachuting-related medically diagnosed injury was reported in the open-text item; otherwise = 0.
- **Substantial problem:** Substantial problem = 1 when an affected region was accompanied by moderate or large reduction in participation or performance, or inability to participate.
- **Temporality:** The questionnaire did not record whether problem onset occurred before or after the night jump(s) with equipment. Temporal order cannot be reconstructed.

## Supplementary Figure S1. Available analytical participant flow

| Available participant-flow stage |
|---|
| Potentially eligible / approached / declined: exact counts not retained in the recruitment log |
| ↓ |
| Analytical cohort with a classifiable primary outcome: n = 105 |
| ↓ |
| Principal Firth model: n = 105; men-only sensitivity: n = 102; physical-test analyses: variable-specific available cases (Table S5) |

## Supplementary Table S7. STROBE cross-sectional reporting checklist

| Item | Topic | Location / reporting status |
|---|---|---|
| 1 | Title and abstract | Cross-sectional design stated in title; abstract states same-window exposure/outcome and temporal limitation. |
| 2 | Background/rationale | Introduction, paragraphs 1-3. |
| 3 | Objectives | End of Introduction; primary exposure and exploratory variables distinguished. |
| 4 | Study design | Methods 2.1. |
| 5 | Setting | Methods 2.1; questionnaire dates and field-assessment month reported. |
| 6 | Participants | Methods 2.2; eligibility reported. Recruitment denominator and refusal count were not retained and this is explicitly disclosed. |
| 7 | Variables | Methods 2.3-2.5; primary exposure/outcome and exploratory variables defined. |
| 8 | Data sources/measurement | Methods 2.3-2.7 and Appendix S1. |
| 9 | Bias | Methods 2.7 and Discussion: convenience/opportunity bias, recall, residual confounding, possible underreporting and outcome-related missingness. |
| 10 | Study size | Methods 2.1; fixed accessible cohort, prevalence precision and events-per-parameter justification. |
| 11 | Quantitative variables | Methods 2.8; continuous variables standardised for adjusted models. |
| 12 | Statistical methods | Methods 2.8; Firth regression, modified Poisson PR model, sensitivity analyses, FDR, missing-data approach and collinearity diagnostics. |
| 13 | Participants | Results and Figure S1. Exact eligible/approached/declined counts unavailable from retained recruitment records. |
| 14 | Descriptive data | Results and Table 1; Supplementary Table S1. |
| 15 | Outcome data | Results and Table 2. |
| 16 | Main results | Results, Table 3, Figures 1-2 and Supplementary Tables S2-S3. |
| 17 | Other analyses | Results and Supplementary Tables S2, S4-S6. |
| 18 | Key results | Discussion opening paragraph. |
| 19 | Limitations | Discussion limitations paragraph; major biases and consequences specified where supportable. |
| 20 | Interpretation | Discussion; causal and temporal claims restricted. |
| 21 | Generalisability | Discussion limitations; single specialised battalion and predominantly male sample. |
| 22 | Funding | Funding statement: no external funding. |

The checklist reports what can be supported by the retained study documentation. Items requiring unrecoverable recruitment counts are marked as unavailable rather than reconstructed.
